# Direct foam writing in microgravity

**DOI:** 10.1038/s41526-021-00185-1

**Published:** 2021-12-21

**Authors:** Guy Jacob Cordonier, Cicely Sharafati, Spencer Mays, Lukas Thackery, Ellena Gemmen, Samuel Cyphert, Megan Brown, John Quinn Napolillo, Savannah Toney, Hunter Moore, John M. Kuhlman, Konstantinos A. Sierros

**Affiliations:** Department of Mechanical and Aerospace Engineering, West Virginia University, Morgantown, WV USA

**Keywords:** Porous materials, Mechanical engineering

## Abstract

Herein we report 2D printing in microgravity of aqueous-based foams containing metal oxide nanoparticles. Such hierarchical foams have potential space applications, for example for in situ habitat repair work, or for UV shielding. Foam line patterns of a TiO_2_-containing foam have been printed onto glass substrates via Direct Foam Writing (DFW) under microgravity conditions through a parabolic aircraft flight. Initial characterization of the foam properties (printed foam line width, bubble size, etc.) are presented. It has been found that gravity plays a significant role in the process of direct foam writing. The foam spread less over the substrate when deposited in microgravity as compared to Earth gravity. This had a direct impact on the cross-sectional area and surface roughness of the printed lines. Additionally, the contact angle of deionized water on a film exposed to microgravity was higher than that of a film not exposed to microgravity, due to the increased surface roughness of films exposed to microgravity.

## Introduction

Currently planned US space exploration missions include manned missions to the Moon and to Mars. One way to significantly reduce mission launch mass and mission cost, which has been studied at least since the 1970s^1,2^, is to manufacture as much of the mission mass as possible in situ, preferably via in situ resource utilization. For example, three-dimensional (3D) printing techniques could be used to fabricate needed parts or technology in situ^3,4^. 3D printing has been shown to be capable of printing a vast array of materials (e.g., hydrogels^5,6^, ceramics^7–9^, metals^10,11^) in applications ranging from biomedical^12,13^ to electrical^14–16^ to construction^17,18^. A particular form of 3D printing known as Direct Foam Writing (DFW) has been used to fabricate 3D hierarchical^19,20^ structures *via* the deposition of foams containing a mixture of oil, water, oxide particles, and other binders^21,22^. Furthermore, foam properties can be tuned through solvent, particle selection, and processing parameters. The present study focuses on single-layer (2D) DFW of oil-in-water foams containing titanium dioxide (TiO_2_), as TiO_2_ is abundant on the Moon^23,24^ and could be used as an ultraviolet (UV) radiation absorber^25,26^ for space vehicle or habitation shielding to protect both astronauts and equipment.

It has been shown that foams in Earth’s gravity with liquid volume fractions (*φ*) between *φ* = 20%–35% are unstable due to the gravitational force causing excess liquid to drain, creating a vertically stratified liquid content profile^27–29^. However, foams formed without the influence of gravity do not undergo this convective drainage, and foams with *φ* = 30% have been demonstrated to be stable in microgravity^30^. A stable foam containing TiO_2_ with variable liquid volume fraction could be useful for performing in situ space vehicle or habitation repair or for UV radiation shielding. To date, there exists a knowledge gap on the ability to 3D print foams in microgravity conditions. The purpose of this study is to demonstrate the ability to perform 2D DFW of a TiO_2_-containing foam in microgravity conditions and perform initial characterization of the foam properties. These initial results could be used in the future to expand printing 3D foam structures in microgravity.

## Results and discussion

### Foam density and liquid volume fractions

Immediately after preparation of the foam, the foam density was measured to be 0.974 (+/−0.02) g/cm^3^, and the foam liquid volume fraction was measured to be 65% (+/−5%).

### Scanning electron microscopy (SEM)

**Fig. 1:**
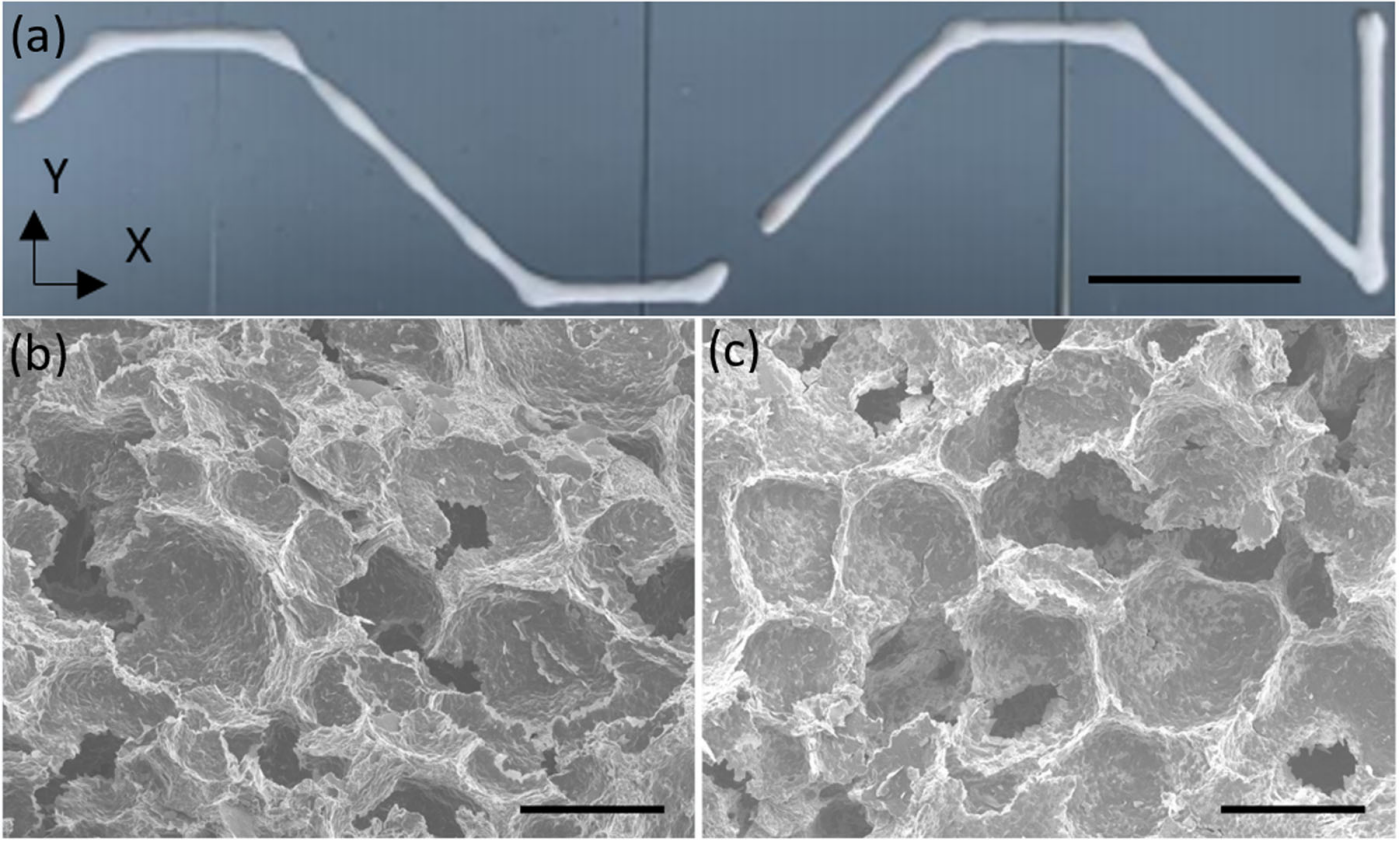
Optical and SEM images of the printed foam. **a** Optical image of the foam immediately after printing on glass slides in microgravity. **b**, **c** Cross-sectional SEM images of the air-dried foams printed in (**b**) microgravity and (**c**) Earth gravity. Scale bars are **a** 25 mm and **b**, **c** 100 μm.

Figure 1a shows an optical image of the printed foam immediately after printing on a glass slide in microgravity during a parabolic aircraft flight. SEM images of the foam printed in microgravity and Earth gravity (Fig. 1b, c, respectively) show that both printed lines (Fig. 1a) exhibit a closed-cell internal structure. Both printed lines were allowed to dry in ambient conditions for several days before analysis. Qualitatively, the morphology of the internal structure of both prints appears to be nearly identical with typical cell size on the order of 100 µm, suggesting the lack of gravitational force during printing does not significantly impact the diameter of the bubbles as the foam is extruded.

### Coarsening

**Fig. 2:**
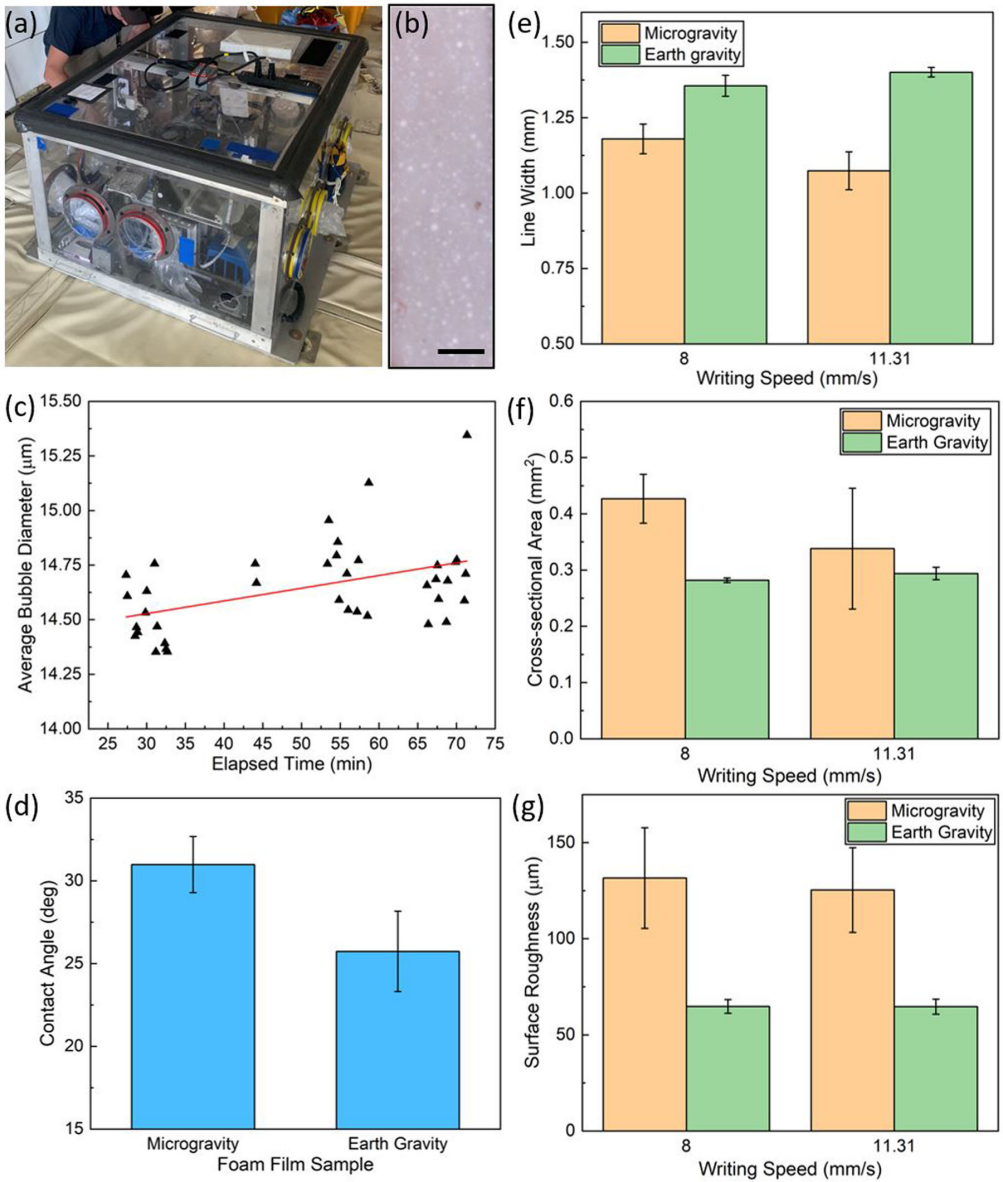
Coarsening, Contact Angle, and Optical Profilometry Plots. **a** Image of the payload containing the experiments onboard the parabolic flight aircraft. Photograph used with permission from Zero Gravity Corporation^32^. **b** Optical image of the foam during the coarsening experiment. Scale bar is 500 μm. **c** Average bubble diameter of the foam over the course of the parabolic flight. **d** Contact angle of DI water on films exposed to microgravity and Earth gravity conditions. (**e**–**g**) Average printed **e** line widths, **f** cross-sectional area, and **g** surface roughness for two writing speeds printed in microgravity and Earth gravity conditions. All error bars represent standard deviation.

Figure 2a shows the payload frame housing the 3D printer (Supplementary Fig. 1) and coarsening experiment on the parabolic flight aircraft. Coarsening tests were conducted to observe and measure the evolution of air bubble diameter within the foam as a function of time, which was prepared by sandwiching a layer of foam between two glass slides. The average apparent bubble diameter of the foam was imaged over the course of the parabolic flight using a digital microscope (Fig. 2b). These average bubble diameters were measured by using an image processing program, ImageJ (National Institute of Health, USA), to calculate the area of each bubble in each image. Then, by assuming each apparent bubble was circular, the diameter was calculated by dividing the area by Pi, taking the square root, and then doubling to value to get the diameter. We note that the pixel resolution of the microscope could limit the accuracy of the measured diameters. It was observed that the bubble diameter increased with time (Fig. 2c), as expected. The smaller bubbles have a higher pressure than the larger bubbles. Due to the surface interactions between nearest neighbors, the gas from the smaller bubbles eventually diffuses into the larger bubbles, decreasing the total count of bubbles, but increasing the average diameter of the remaining bubbles^31^. Using the methodology described by Kennedy et al., the theoretical and experimental effective coarsening rates (D_eff,t_ and D_eff,e_, respectively) of the foam were calculated. D_eff_ is the effective diffusivity of gas between bubbles. D_eff,t_ was found to be 9.75*10^−7^ cm^2^/s. D_eff,e_ was found to be 4.01*10^−11^ cm^2^/s, a significantly lower rate than theory would predict. Several factors could explain this discrepancy. The present foam has a high liquid volume fraction (65%), which should slow down the coarsening rate compared to drier foams. Estimates were made for some parameters, notably film thickness and Henry’s Law constant, which could not be measured in situ. Another source of error could be the transient gravitational effects of the microgravity flight (Each period of microgravity was followed by a period of hypergravity. See the methods section for more detail.), which could impact the drainage of the foam.

### Contact Angle

A difference in contact angle was observed for deionized (DI) water between thin films (thickness = 1.4 mm) exposed to microgravity and Earth gravity (Fig. 2d). An average angle of 30.98 ± 1.70° was observed for the film that was printed in microgravity, compared to an average angle of 25.74 ± 2.43° for the Earth gravity film; this lower angle is expected to increase lateral spreading of the printed foam lines.

### Foam line widths, cross-sectional area, and surface roughness

The foam was printed in both microgravity and Earth gravity (in the laboratory), and at two different speeds, 8 and 11.31 mm/s. The printed foam line from one microgravity parabola is illustrated in Fig. 1a. The horizontal and vertical line segments in the image were printed at 8 mm/s, and the angled line segments (45° relative to the horizontal and vertical segments) were printed at 11.31 mm/s. The average line widths were measured to be 1.18 ± 0.05 mm and 1.07 ± 0.09 mm for the horizontal/vertical and angled line segments, respectively, using optical profilometry for the microgravity print (Fig. 2e). The average line widths for the Earth gravity print were measured to be 1.36 ± 0.04 mm and 1.40 ± 0.02 mm for the horizontal/vertical and angled line segments, respectively. The extrusion pressure remained constant at 20.7 kPa for all line segments. Each line was printed with a standoff distance of 0.220 mm. For the microgravity case, the thinner line width at a faster writing speed is consistent with conservation of mass: the foam volume printed per unit time remained the same, but the writing distance increased per unit time. There was less material per unit distance printed, leading to a decrease in the spread of the foam upon deposition on the glass substrate. However, it is noted that the results for the Earth gravity case are not statistically distinguishable from each other (i.e., their respective error bars overlap). The line widths for both writing speeds in Earth gravity are higher than their respective line widths in microgravity. This could indicate that the gravitational force does play a role in the spreading of the foam. This is probably related to the interfacial surface tension between the entrapped air, foam walls, and the glass substrate. Without gravity, surface tension will cause the bubbles in the foam to attempt to become more spherical; with gravity, they will become more deformed. This deformation, relative to the foam in microgravity, may cause the foam to spread on the substrate more readily at higher writing speeds. We suspect that within a few seconds after deposition, the foam becomes “pinned” to the substrate, preventing further spreading and deformation during the hypergravity portion of the flight parabola.

Cross-sectional area (Fig. 2f) and surface roughness (Fig. 2g) were also measured using optical profilometry. Interestingly, the cross-sectional area was higher for both writing speeds in microgravity compared to Earth gravity (51.4% and 15.1% increase for the 8 and 11.31 mm/s lines, respectively). Coupling this information with the results from the line width measurements, it can be inferred that the lack of gravity during the extrusion allows the foam to better retain its shape after printing. It does not spread as readily on the glass substrate in microgravity as in Earth gravity. The surface roughness exhibited a similar trend. The foam line segments printed in microgravity have a higher measured surface roughness (103% and 93.9% increase for the 8 and 11.31 mm/s lines, respectively) than the segments printed in Earth gravity. We attribute this difference in surface roughness to the shape of the profile of the printed lines, as stated above.

## Conclusion

This work has demonstrated that gravity plays a significant role in the process of DFW. The foam spread less over the substrate when deposited in microgravity as compared to Earth gravity. This had a direct impact on the cross-sectional area and surface roughness of the printed lines, as well, increasing both values. Additionally, the contact angle of DI water on a film exposed to microgravity was higher than that of a film not exposed to microgravity. Additional work in this area should focus on exploring applications that can take advantage of these results and expand the experimental design to fabricate 3D foam structures in microgravity. Hierarchical foams have potential for in space use for in situ habitat repair work or UV shielding.

## Methods

### Flight profile and gravitational acceleration over time

This flight took place on a modified Boeing 727–200 (operated by Zero Gravity Corporation, “Zero-G”). Microgravity conditions were achieved by flying a series of 25 parabolic microgravity flight paths. The foam line in Fig. 1a was printed in a 20 s time segment in which the plane flew downwards until the plane’s vertical acceleration matched that of Earth’s gravitational acceleration, 9.81 m/s^2^, simulating microgravity (Supplementary Fig. 2). Each microgravity parabola was followed by a short period of hypergravity (gravitational acceleration between 1- and 2-times Earth gravity) as the plane pulled up.

### Metal oxide-stabilized foam preparation

The foam line shown in Fig. 1a was printed using a foam formulation that was prepared by combining aqueous and oil liquid phases and frothing to entrap air^21,22^. The aqueous phase was prepared by combining 2.87 g of titanium dioxide particles (21 nm diameter, Aeroxide P25, Aldrich), 6.48 g of deionized (DI) water, and 1.77 g of titanium(IV) bis(ammonium lactato) dihydroxide solution (TALH, 50 wt. % in water, Aldrich) in a 50 mL beaker. The molar ratio between TiO_2_ particles and TALH was kept at 12:1. The mixture was stirred with a magnetic stir rod for 15 min at 350 rpm, followed by sonication in an ice water bath for 15 min. This stirring-sonication step was carried out once more before a final stirring for 24 h. The oil phase was prepared by combining 0.85 g of stearic acid (97%, Acros Organics), 1.18 g of polysorbate 60 (Alfa Aesar), and 0.8 g of glycerol (>99%, Sigma-Aldrich) in a 250 mL beaker. The molar ratio between TALH and stearic acid was kept at 1:1. The mixture was stirred with a magnetic stir rod on a hot plate at 80 °C for 5 min, melting the stearic acid. Next, the mixture was stirred at 350 rpm for 5 min, homogenizing the constituents. The aqueous phase was then incorporated into the oil phase using a pipette while continuing to stir and heat. The mixture was stirred and heated until becoming homogenous. Finally, the mixture was removed from the stirring hot plate and frothed at 1,500 rpm for 8 min with a tabletop overhead stirrer with an impeller attachment, entrapping air and forming the foam.

To prepare the foam for transportation to the Zero-G aircraft, it was transferred to 3 cc syringe barrels (Nordson) which were then sealed. A plunger was placed on top of the foam inside the barrel to aid in evenly distributing the air pressure for extrusion. Once ready to use in flight, the seals were removed and a plastic tapered nozzle tip (Nordson) of inner diameter 0.58 mm was attached to each syringe. The foam was extruded through the nozzle only during the microgravity portions of each parabola during the flight onto microscope glass slide substrates. The glass substrates were cleaned, first with detergent in DI water, and then isopropyl alcohol before loading into the payload.

### 3D printer design and operation

Six sets of four glass microscope slide substrates were mounted in custom plastic substrate holder frames that were secured to the 3D printer (Supplementary Fig. 1) via two parallel T-slot quarter-round rails mounted on translatable *XY*- and *Z*-stages (Applied Scientific Instrumentation, ASI). The *XY*-stage moved the substrates relative to the nozzle and the *Z*-stage controlled the nozzle’s standoff distance to the substrates. An ASI control box (LX-4000) was used to send custom code from a laptop to the stages to perform the 3D printing. Micro-Manager (ver. 1.4) software (together with the MMCorePy Python library) was used to communicate with the control box. In Python, coordinates for each print beginning and ending location were referenced from pre-written Excel files; then the coordinate commands were sent to the ASI Control Box. The entire assembly was housed in a payload secured to the floor of the aircraft during flight (Fig. 2a)

To command pressure to the nozzle, the Python code sent binary data to the serial monitor, where a Teensy 4.0 microcontroller (3DmakerWorld) was reading the data via the C++ Arduino platform. Printing would begin whenever a SparkFun 3-axis accelerometer (Model MMA8451) connected to the Teensy 4.0 detected that the *Z*-direction (i.e., gravitational) component of acceleration was 2.94 m/s^2^ or less. Upon seeing a ‘1’, the Teensy set a general-purpose input/output pin (GPIO) high, which sent a 5 V signal to the Nordson Ultimus II EFD pressure controller to apply the pre-set extrusion pressure to the syringe. A Kobalt 2 Gallon Quiet Tech air compressor (Model 3300243) supplied the high-pressure air required to operate the Nordson EFD to extrude the foam. A ‘0’ was sent by the Python code to the serial monitor at the end of each parabola to stop the 3D printer traverse once the *Z*-direction acceleration was above 2.94 m/s^2^; this command also caused the Nordson EFD pressure controller to stop applying pressure to the syringe.

The standoff distance between the nozzle tip and the glass substrates was measured to be 0.220 mm, taken immediately prior to the deposition during flight using a feeler gauge. The extrusion pressure was set to 20.7 kPa. The writing speed was 8 mm/s for the horizontal and vertical foam line segments, and 11.3 mm/s for the diagonal segments, when both the *X-* and Y-axis motors were driven in conjunction (Fig. 1a).

### Characterization

Scanning Electron Microscopy (SEM) was performed using a JEOL JSM-7600F scanning electron microscope operated with a 5.0 kV bias and a working distance of 10 mm. To preserve the structure of the foam as printed, samples were not treated with any post-processing or curing steps beyond air-drying. A razor blade was used to cut a cross-section of each printed foam sample, which was then placed on copper tape. The exposed cross-sections were sputtered with gold-palladium before observing in the SEM.

The contact angle was determined by measuring images of sessile drops of deionized water on a flat film of the foam. One film was exposed to zero gravity aboard the parabolic flight. The other film remained in Earth’s gravity. The droplets were 2 μL in volume each and deposited using a Matrix Electronic Pipette (Thermo Scientific). Images were taken with a digital microscope and analyzed using ImageJ to measure the angles.

A Bruker Contour GT K0 Optical Profilometer with a green light source was used to measure line width, cross-sectional area, and surface roughness of the horizontal and vertical line segments of the foam line (Fig. 1a). A total of 32 lateral thickness scan measurements were taken for each of the line segments.

For the coarsening experiment, doctor blading was used to coat a clean glass slide with the foam. The thickness was set at 1,400 μm. A second glass slide was placed on top of the foam and then taped to it, to prevent any relative motion of the slides, which could obfuscate the time evolution of the bubbles. The assembly was loaded onto the payload with a fixed digital microscope focused on the bubbles. A laptop interfaced to the microscope recorded images at 10 s intervals over the flight duration.

Foam density was measured by measuring foam volume and foam mass in each syringe. Foam air volume fraction was measured by measuring the prepared ink volume before and after frothing.

### Reporting summary

Further information on research design is available in the Nature Research Reporting Summary linked to this article.

## Supplementary information

Direct Foam Writing in Microgravity - Supplemental Information

Reporting Summary

## Data Availability

The authors declare that the data supporting the findings of this study are available within the article and its supplementary information file and are available from the corresponding author upon reasonable request.
