# Supplementary material for: Direct foam writing in microgravity
Source: NPJ Microgravity. 2021 Dec 21;7:55. doi: 10.1038/s41526-021-00185-1 (PMC8692601; doi:10.1038/s41526-021-00185-1)
Supplement: Supplementary file 1 — Direct Foam Writing in Microgravity - Supplemental Information [file 41526_2021_185_MOESM1_ESM.pdf]

Direct Foam Writing in Microgravity

Supplementary Information

Supplementary Figures

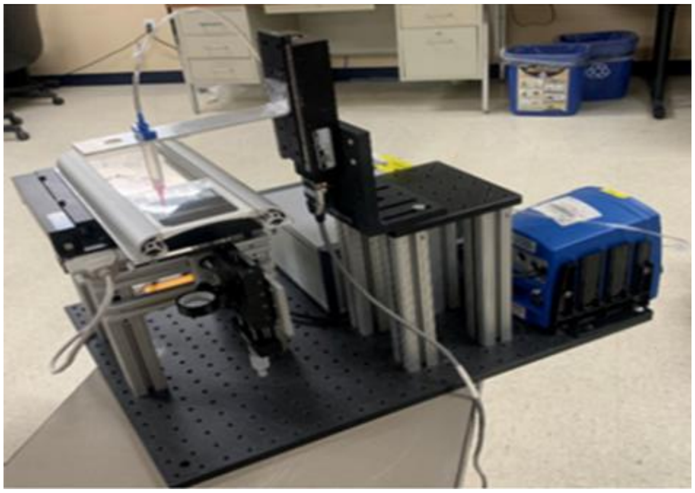

Supplementary Figure 1. Image of the 3D printer used to deposit the foam in microgravity and earth gravity. The printer is housed in the payload shown in Fig. 2a.

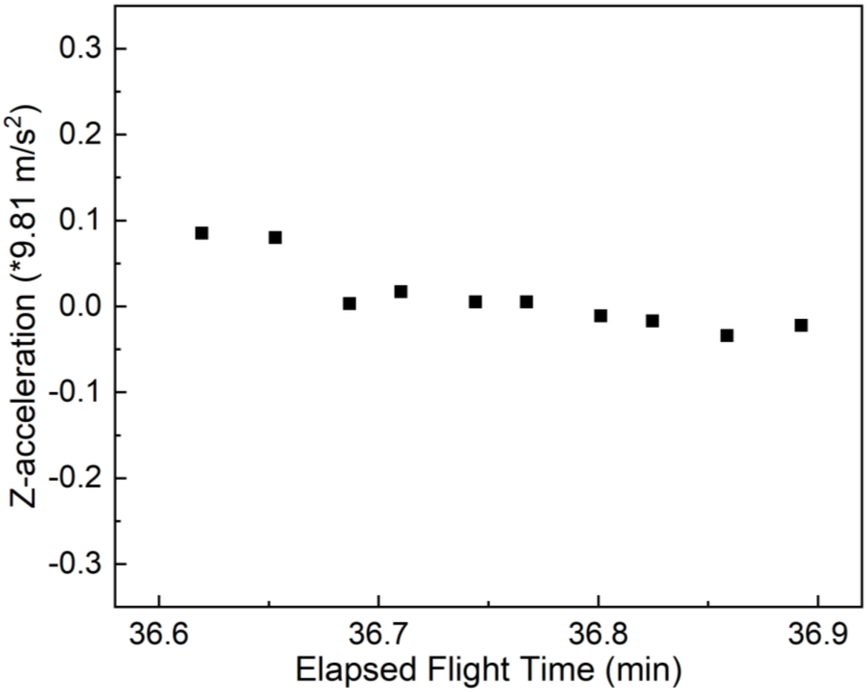

Supplementary Figure 2. Measured acceleration in the z-direction versus elapsed flight time. The foam line in Fig. 1a was printed during this period of microgravity.
